# Nicotine absorption from electronic cigarette use: comparison between experienced consumers (vapers) and naïve users (smokers)

**DOI:** 10.1038/srep11269

**Published:** 2015-06-17

**Authors:** Konstantinos E. Farsalinos, Alketa Spyrou, Christos Stefopoulos, Kalliroi Tsimopoulou, Panagiota Kourkoveli, Dimitris Tsiapras, Stamatis Kyrzopoulos, Konstantinos Poulas, Vassilis Voudris

**Affiliations:** 1 Onassis Cardiac Surgery Center, Athens, Greece; 2 Department of Pharmacy, University of Patras, Rio, Greece

## Abstract

Electronic cigarettes (ECs) are nicotine delivery devices that are proposed as tobacco harm reduction products to smokers. Nicotine delivery from ECs is potentially important in their efficacy as smoking substitutes. Herein, nicotine delivery from using a new-generation EC device (variable-wattage, set at 9 W) was evaluated, comparing experienced (vapers) with naïve users (smokers). Twenty-four vapers and 23 smokers participated to the study. They were asked to obtain 10 puffs in 5 minutes and then use the EC ad lib for 60 more minutes (total duration of use: 65 minutes). An 18 mg/mL nicotine-containing liquid was used. Blood samples were obtained at baseline, 5-minutes and every 15 minutes thereafter, while number of puffs and average puff duration were recorded. Although at baseline both groups had similar plasma nicotine levels, smokers consistently exhibited lower levels at all time-periods; at 5-minutes the levels were lower by 46%, while during the subsequent period they were lower by 43% (at 65-minutes) to 54% (at 20-minutes). Both groups took similar number of puffs, but smokers had average puff duration of 2.3 s compared to 3.5 s in vapers. Even in vapers, plasma nicotine levels at 5 minutes were lower than those observed after smoking 1 tobacco cigarette.

Electronic cigarettes (ECs) have been introduced to the market in recent years as an alternative to smoking and part of tobacco harm reduction. Awareness and use of these products is growing exponentially^1^. Their popularity could be attributed to the fact that they deal both with the chemical, through nicotine delivery, and the psycho-behavioral part of addiction to smoking^2^. However, the potential of ECs to deliver nicotine to the user has not been adequately assessed. Initial studies showed minimal nicotine absorption; however, the researchers recruited smokers with no experience with EC use, while the devices used are currently considered as outdated^3^,^4^. Studies recruiting experienced users (commonly called “vapers”) showed that substantial amounts of nicotine can be absorbed^5^,^6^. Nicotine intake may also be influenced by the type of EC device used, with a recent study showing that new-generation devices, providing high power to the atomizer for aerosol production, can deliver nicotine faster and more efficiently^7^.

It has been shown that there is a substantial difference in vaping topography patterns between experienced and naïve EC users^8^,^9^. Smokers are used to inhaling from a cigarette that is already burning, while EC use is associated with aerosol production only at the time of activation. This can cause a substantial delay between activation and production of sufficient amount of vapor; experienced users compensate this by activating ECs for longer time, taking longer puffs^8^. Moreover, while smokers can draw “harder puffs” (i.e. can elevate the puff volume which will accelerate the burning process and produce more smoke without increasing the puff duration), such a pattern has no effect on aerosol production from ECs. Therefore, it is expected that different use patterns will result in different nicotine pharmacokinetics. The purpose of this study was to evaluate the nicotine delivery potential of a new-generation EC devices in a group of smokers with no previous experience in EC use compared to a group of experienced vapers, and to examine the association between nicotine absorption and puffing topography.

## Methods

### Study participants

Experienced vapers and smokers without any previous experience with EC use were recruited in this study. To participate, vapers had to be former daily smokers who had quit smoking and were using ECs daily for at least 1 month; smokers had to be smoking daily for at least 5 years and should have never used an EC before. Other inclusion criteria for both groups were: (1) age 18 to 60 years; (2) being clinically healthy, with no history of cardiovascular and lung disease or hematological problems; (3) able to remain abstinent from EC use and smoking for at least 8 hours; and (4) willing to provide blood samples. Exclusion criteria were: (1) Fainting or feeling faint when providing blood samples; (2) pregnancy; and (3) unwillingness to provide written informed consent. The study was performed in accordance with the Declaration of Helsinki ethical principles for medical research involving human subjects. The protocol was approved by the ethics committee of Onassis Cardiac Surgery Center, and written informed consent was provided and signed by all subjects before participating to the study. No financial or other compensation was provided to the participants.

### Materials and clinical procedure

**Figure 1:**
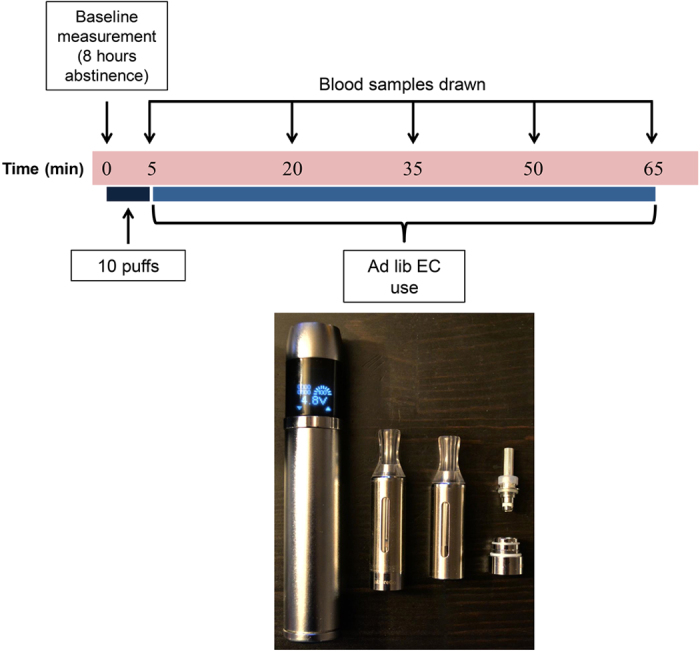
Protocol and materials (electronic cigarette device and atomizer) used in the study.

An advanced, new-generation EC device was used in this study (Fig. 1). The device (EVIC, Joyetech, Shenzhen, China) consists of a high-capacity rechargeable lithium battery (2600 mAh) with an internal electronic circuit which includes a current stabilizer and allows the user to manually adjust the power (W) applied to the atomizer. The energy delivery to the atomizer was set at 9 W. The battery was fully charged before use. A new-generation atomizer (EVOD, KangerTech, Shenzhen, China) was used and was filled with approximately 2 ml of liquid. An 18 mg/ml nicotine-containing liquid was used (Max Blend, Flavourart SRL, Oleggio, Italy). The liquid was analyzed for nicotine concentration and the presence of contaminants, with the results shown in Table 1. Smokers were instructed that the activation button needs to be continuously pressed during the puff. No other instructions or advice on the use-patterns of the EC were given.

Participants visited the laboratory after abstaining from EC use and smoking, caffeine, alcohol and food intake for at least 8 hours. The Fagerström Test for Cigarette Dependence (FTCD)^10^ and the Cigarette Dependence Scale (CDS)^11^ were provided to all participants in order to assess current (for smokers) and past (for vapers) smoking dependence. Additionally, the two questionnaires were modified in order to assess EC use dependence in vapers, as described previously^7^. In brief, the questions related to tobacco cigarette consumption were substituted with EC liquid consumption, based on the results of a large survey of 19,441 vapers. The consumption was classified according to percentiles (quartiles for FTCD and quintiles for CDS). The results of the two tests after excluding the question on cigarette and EC consumption were also reported (as FTCD-modified and CDS-modified, Table 1).

A venous catheter was introduced in an antecubital vein and 8 ml of venous blood was collected in lithium-heparinized vacutainers to measure baseline plasma nicotine levels. Participants were asked to take 10 puffs in 5 minutes, simulating tobacco cigarette use^12^. Subsequently, they were asked to use the ECs ad lib for an additional period of 60 minutes (total duration of use: 65 minutes). Venous blood samples were obtained after the 5-minute period and every 15 minutes during the subsequent 60-minute period.

The blood samples were stored in ice and were centrifuged within 1 hour. Plasma was separated and stored at −70 °C until analyzed. Measurements of nicotine levels were performed in a specialized laboratory by Gas Chromatography with an NPD-80 Specific Detector. The lowest limit of quantification (LOQ) for this method was 0.5 ng/ml. For samples with nicotine levels below the LOQ, a value of LOQ/2 was assigned for statistical analysis.

### Puffing topography assessment

A characteristic of the EC device used in the current study is the ability to (a) record the time of activation of each puff and (b) store the puff number, duration and time of each puff in internal memory. Subsequently, the data were downloaded with specialized software (provided by the manufacturer of the device) and stored in Excel tables. The number of puffs and average puff duration was calculated from these data. The accuracy of the recorded data was validated in a pilot study, in which 5 vapers were asked to use the device for 30 puffs. The puff number was manually counted and compared with the recorded number of puffs. Additionally, the accuracy of puff duration recording was validated by video-recording 5 puffs per participant and comparing the durations between video-recording and device record.

### Statistical analysis

The distribution of data was assessed by the Kolmogorov-Smirnov test. Categorical variables were expressed as number (percentage) while continuous variables as mean (SEM). The baseline characteristics were compared between groups by using χ^2^ test and student’s t-test. To compare nicotine levels, repeated measures analysis of variance (ANOVA) was used, with 6 timing levels as within-subject variable and two groups (smokers vs. vapers) as between-subject factor. To compare puffing patterns (number and duration of puffs), student’s t-test was used. The association between the elevation in nicotine levels from baseline to 65-minute timing, puffing patterns and smoking history was assessed by using Pearson correlation coefficient. A multivariate linear regression analysis was performed, to assess which factors were associated with a higher elevation in plasma nicotine levels. Change in plasma nicotine levels from baseline to 65 minutes was the independent variable and age, gender, smoking duration and consumption, FTCD and puff duration were introduced to the analysis as covariates. A two-tailed P value of ≤0.05 was considered statistically significant. All analyses were performed with commercially available statistical software (SPSS v.18, Chicago, Illinois, USA).

## Results

Fifty subjects participated to the study. Three of them were excluded from the analysis (2 smokers and 1 vaper) because of inability to obtain blood samples at all timing points. The baseline characteristics of the participants are shown in Table 2. Smokers reported less cigarette consumption compared to vapers. According to the CDS, past smoking dependence was higher in vapers compared to current dependence in smokers; no difference was observed in the FTCD. Additionally, vapers reported lower dependence to EC use compared to their past dependence on smoking.

### Plasma nicotine levels

**Figure 2:**
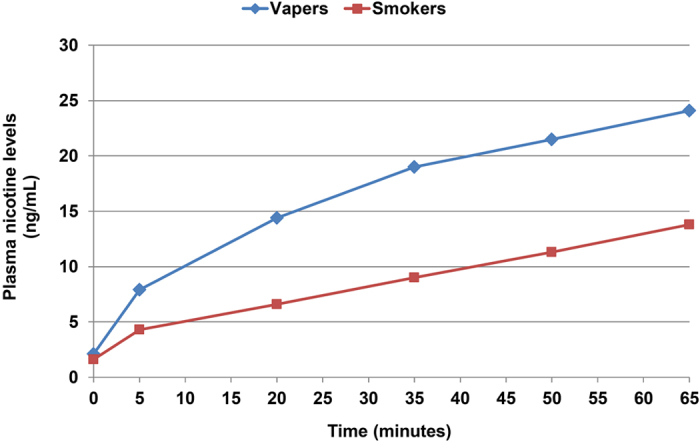
Plasma nicotine levels in experienced (vapers) and naïve users (smokers). No statistically significant difference was observed at baseline. After initiation of electronic cigarette use, there was a statistically significant difference between groups at all time-points (P = 0.020 at 5 minutes, P < 0.001 at all other time points).

The results of plasma nicotine level measurements are shown in Fig. 2. A significant difference was observed between different time points (F = 115.1, P < 0.001) and a significant time x group interaction (F = 11.3, P < 0.001) was observed from repeated measures ANOVA. No difference in plasma nicotine levels was observed at baseline between vapers and smokers. At 5 minutes, plasma nicotine levels rose from 2.1 ± 0.3 ng/mL to 7.9 ± 0.9 ng/mL in vapers (P < 0.001 compared to baseline) and from 1.6 ± 0.3 ng/mL to 4.3 ± 0.7 ng/mL in smokers (P < 0.001 compared to baseline). At 65 minutes, the levels reached to 24.1 ± 2.0 ng/mL in vapers and to 13.8 ± 1.6 ng/mL in smokers. Large inter-individual differences were observed; plasma nicotine levels at 5 minutes ranged from 1.5 ng/mL to 21.8 ng/mL in vapers and from 1.3 ng/mL to 15.2 ng/mL in smokers. Plasma nicotine levels at 65 minutes ranged from and 10.1 ng/mL to 48.1 ng/mL in vapers and from 3.3 ng/mL to 31.4 ng/mL in smokers. At all time-points besides baseline, the difference in plasma nicotine levels between the two groups was statistically significant (P = 0.002 at 5 min, P < 0.001 at all other time-points), with vapers consistently having higher plasma nicotine levels.

### Puffing patterns and predictors of plasma nicotine levels

**Figure 3:**
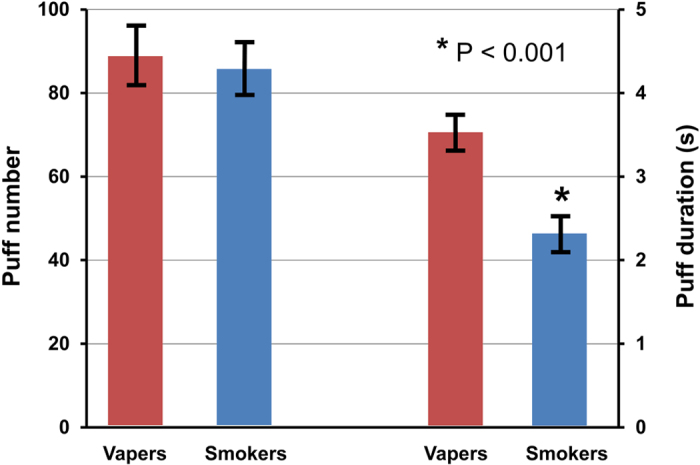
Average puff number and duration of the two groups. No difference was observed in puff number between groups, however, vapers took longer puffs (3.5 ± 0.2 s) compared to smokers (2.3 ± 0.2 s).

The average number of puffs and puff duration in the two study groups are displayed in Fig. 3. Both groups took similar number of puffs within the 65-minute period. However, puff duration was 3.5 ± 0.2 s in vapers, ranging from 1.8 s to 6.2 s and 2.3 ± 0.2 s in smokers, ranging from 0.9 s to 4.3 s (P < 0.001).

A weak but significant correlation was observed between the change in plasma nicotine levels from baseline to 65 minutes and puff duration (r = 0.37, P = 0.01). Significant correlations were also observed between changes in plasma nicotine levels from baseline to 65 minutes and cigarettes smoked per day (r = 0.41, P = 0.005), FTCD (r = 0.41, P = 0.004) and CDS (r = 0.36, P = 0.013). Puff duration was the only factor which correlated with changes in plasma nicotine levels from baseline to 5 min (r = 0.31, P = 0.036). In a multivariate linear regression analysis, only puff duration (β = 0.37, P = 0.008) and cigarette consumption (β = 0.38, P = 0.005) were significantly associated with the change in plasma nicotine levels from baseline to 65 minutes.

## Discussion

This is the first study to directly compare nicotine delivery from EC use between experienced and naïve EC users. A new-generation device was used, which has been previously shown to deliver nicotine more effectively compared to first-generation (cigarette-like) devices^7^. The study clearly showed that both groups obtain nicotine from EC use, however, faster absorption rate and higher plasma nicotine levels were observed in experienced compared to naïve users. It seems that this is partly explained by differences in puffing patterns between the two groups; in particular, smokers took shorter puffs compared to vapers, and puff duration was independently associated with the elevation of plasma nicotine levels after 65 minutes of use.

New generation EC devices are predominantly used by dedicated users who, in most cases, are heavy ex-smokers^2^. The hypothesis that such devices deliver nicotine more efficiently compared to first-generation devices was confirmed in a recent clinical study which found higher plasma nicotine levels in experienced users when using a new-generation device compared to a “cigarette-like” product^7^. Subsequently, a laboratory study confirmed that elevating power levels leads to increased aerosol yield and nicotine delivery from the liquid to the aerosol^13^. Herein it was shown that naïve users are unable to obtain similar levels of nicotine compared to experienced users when using advanced devices. This was partly explained by differences in puff duration between the two groups. Smokers were using the EC in a similar puffing pattern as tobacco cigarettes, taking shorter puffs compared to experienced users. Similar observations were reported in a previous study of EC use topography^8^. In that study, vapers took 4.2 sec puffs, compared to 2.4 sec puffs observed in naïve users. In the present study vapers took somewhat shorter puffs, which can be explained by the higher EC power used in this study. However, considering the weak correlation between changes in plasma nicotine levels and puff duration, it seems that additional factors contribute to nicotine absorption. Such factors could be depth of inhalation and time of keeping the vapor inhaled.

Smoking is a difficult addiction to break. Tobacco cigarettes deliver nicotine very effectively, mostly in terms of speed of absorption^14^. Speed of delivery, as well as other substances in smoke which potentiate the addictive properties of nicotine^15^, contribute significantly to the dependence potential of tobacco cigarettes. It is reasonable to assume that alternative products would be more successful in substituting smoking if they could replicate the nicotine delivery potential of tobacco cigarettes. Randomized controlled studies evaluating the efficacy of ECs in substituting smoking have shown modest results^16^,^17^. One of the main reasons for this was assumed to be the low potential of the devices used in those studies to deliver nicotine to the user. Despite the fact that new-generation devices are more efficient, they still lag behind in speed of nicotine delivery compared to smoking, which typically raises plasma nicotine levels to 15 ng/mL or more within 5 minutes^14^. In naïve users, the lower potential of ECs to deliver nicotine is more prominent. A recent study showed enhanced nicotine intake after using first-generation EC device for 4 weeks^5^. By assessing patterns of use, our study confirms previous observations that there is a learning curve in the use of ECs, even when new-generation devices are used; experienced users use ECs more intensively compared to novice users^8^,^17^. Since less than 20% of smokers who try ECs progress to using them daily^18^,^19^, our results indicate that smokers need to be properly informed about the difference in characteristics of EC use compared to tobacco cigarettes in order to avoid any initial disappointment which would discourage further use and compromise successful smoking substitution or lead to dual use of tobacco cigarettes and ECs.

Some limitations apply to this study. Vapers used different equipment and liquid from what they were regularly using. It is possible that, by being more familiar, vapers would get higher plasma nicotine levels when using their own equipment. Moreover, we did not provide participants with a choice of different flavours, thus it is possible that some of them underused the EC because they did not like the taste of the liquid used in the study. All blood samples were obtained immediately after use, without any period of abstinence; therefore, we were unable to determine whether there is any delayed nicotine absorption, which would indicate absorption through the oral mucosa. However, a study evaluating first generation ECs showed no delayed absorption^5^; most likely, the same applies to use of newer-generation devices. Only puff number and duration was measured to assess patterns of use. In smoking, puff volume is also important in terms of nicotine delivery. However, Talih *et al.* found no effect of puff volume on nicotine delivery to the aerosol of ECs^13^. The potential of ECs to deliver nicotine within 5 minutes may have been underestimated due to the fact that participants were informed that they could vape ad lib for 65 minutes overall during the experimental session. If the protocol dictated EC use for 5 minutes only, with a subsequent abstinence period, it is possible that participants would have used the devices more intensively since they would have little time to satisfy their nicotine need, resulting in higher plasma nicotine levels. Moreover, they were not allowed to take more than 10 puffs during that period; ad lib use could have resulted to higher number of puffs taken during the 5 minute period, which could influence plasma nicotine levels. An 18 mg/mL nicotine containing solution was used in this study. Although it is possible to obtain and use liquid with higher levels of nicotine in the US, the European Union Tobacco Product Directive dictates a maximum limit of 20 mg/mL nicotine concentration in EC liquids sold within the European Union. This will be implemented from 2016 and is applicable to all products besides those which will obtain medicinal license; currently, no EC product has got medicinal license, due to the long-lasting and very expensive process. Our study indicates that this limit may be inadequate to fully satisfy the needs of smokers in terms of nicotine intake. Previous studies have shown that a substantial proportion of smokers need to use high nicotine-containing liquids in order to quit smoking^2^. However, the evolution of new products might result in better nicotine delivery from the liquid to the aerosol and faster nicotine absorption in the future, without the need to use higher nicotine concentration liquids. Although this could make ECs more addictive, it raises an important ethical issue of whether a product, which is probably beneficial for a part of the population (smokers), should be restricted (which could result in reduced efficacy as a smoking substitute) because some other parts of the population (non-smokers) decide to voluntarily adopt its use and perhaps becoming addicted to it^20^. Any regulation should be based on the assessment of the balance between any harm from use by non-targeted (non-smokers) versus the potential benefits from use by targeted population groups (smokers). More population studies are needed to assess this aspect, however, it is important to properly educate the society that ECs should be used as smoking substitutes and not as a new healthy habit for anyone to adopt. Finally, the equipment used in this study, although considered new-generation, is already outdated. EC technology is progressing at a fast pace, and research is sometimes unable to follow this progress and assess the efficacy of such devices promptly. The battery device we used has been now substituted with a newer version generating more power, while newer-generation atomizers can withstand higher power levels and puff durations without resulting in the unpleasant taste associated with the dry-puff phenomenon^8^. This can lead to higher production of vapor and, thus, higher nicotine content in the aerosol of each puff.

In conclusion, new-generation ECs can effectively deliver nicotine, but at a slower rate and at lower levels in naïve compared to experienced users. Users should be properly informed at the initiation of EC use that nicotine intake will likely increase as they learn and adjust their use-patterns. Faster nicotine delivery is expected to increase the appeal of ECs to smokers and could make them more effective as smoking cessation tools, at the cost of potentially increasing their dependence potential.

## Additional Information

**How to cite this article**: Farsalinos, K. E. *et al.* Nicotine absorption from electronic cigarette use: comparison between experienced consumers (vapers) and naïve users (smokers). *Sci. Rep.* **5**, 11269; doi: 10.1038/srep11269 (2015).

## Figures and Tables

**Table 1:** Analysis of the electronic cigarette liquid used in the study.

Analysis	Quantity
Nicotine	17.7 mg/ml
Propylene glycol	389 mg/ml
Glycerol	751 mg/ml
Diethylene glycol	ND
Aldehydes (total)	18.17 μg/ml
Acetaldehyde	8.51 μg/ml
Crotonaldehyde	6.33 μg/ml
Formaldehyde	3.33 μg/ml
Diacetyl	ND
Tobacco-Specific Nitrosamines (total)	2.08 ng/ml
NNN	ND
NNK	2.08 ng/ml
Heavy metals (total)	35 ng/ml
Arsenic	35 ng/ml
Chromium	ND
Lead	ND
Nickel	ND
pH	8.55
Water	4.3%

Abbreviations. ND, not detected; NNN, N′-nitrosonornicotine; NNK, 4-(methylnitrosamino)-1-(3-pyridyl)-1-butanone.

**Table 2:** Baseline characteristics of study participants.

Characteristic	Vapers (n=24)	Smokers (n=23)	P
Males, n (%)	18 (75.0)	14 (60.9)	0.299
Age, years	39.6 (1.9)	39.5 (1.9)	0.982
Smoking duration, years	21.0 (1.9)	21.2 (2.0)	0.949
Smoking consumption, cigarettes per day	33.9 (2.4)	25.0 (1.2)	0.002
EC use duration, months	19.4 (2.3)		
Smoking cessation duration, months	18.7 (2.3)		
FTCD smoking	7.0 (0.4)	6.0 (0.5)	0.117
FTCD smoking-modified^a^	4.7 (0.30)	4.3 (0.4)	0.412
CDS smoking	53.4 (1.1)	47.3 (1.7)	0.005
CDS smoking-modified^a^	49.1 (1.0)	43.6 (1.7)	0.006
FTCD-EC	6.09 (0.32)		0.020^b^
FTCD-EC-modified^a^	3.91 (0.27)		0.020^b^
CDS-EC	45.13 (1.51)		<0.001^b^
CDS-EC-modified^a^	41.27 (1.46)		<0.001^b^

^a^Score by subtracting the question about cigarette and EC consumption.

^b^P < 0.05 compared to the respective scores for smoking (paired-samples t-test).
