# Corrigendum: Nicotine absorption from electronic cigarette use: comparison between experienced consumers (vapers) and naïve users (smokers)

**DOI:** 10.1038/srep13506

**Published:** 2015-09-04

**Authors:** Konstantinos E. Farsalinos, Alketa Spyrou, Christos Stefopoulos, Kalliroi Tsimopoulou, Panagiota Kourkoveli, Dimitris Tsiapras, Stamatis Kyrzopoulos, Konstantinos Poulas, Vassilis Voudris

Scientific Reports **5**: Article number: 1126910.1038/srep11269; published online: 06172015; updated: 09042015

This Article contains a typographical error in the Methods section under the subheading ‘Materials and clinical procedure.

“The results of the two tests after excluding the question on cigarette and EC consumption were also reported (as FTCD-modified and CDS-modified, Table 1).”

should read:

“The results of the two tests after excluding the question on cigarette and EC consumption were also reported (as FTCD-modified and CDS-modified, Table 2).”

